# Twenty years of West Nile virus spread and evolution in the Americas visualized by Nextstrain

**DOI:** 10.1371/journal.ppat.1008042

**Published:** 2019-10-31

**Authors:** James Hadfield, Anderson F. Brito, Daniele M. Swetnam, Chantal B. F. Vogels, Ryan E. Tokarz, Kristian G. Andersen, Ryan C. Smith, Trevor Bedford, Nathan D. Grubaugh

**Affiliations:** 1 Vaccine and Infectious Disease Division, Fred Hutchinson Cancer Research Center, Seattle, Washington, United States of America; 2 Department of Epidemiology of Microbial Diseases, Yale School of Public Health, New Haven, Connecticut, United States of America; 3 Department of Pathology, Microbiology and Immunology, University of California, Davis, Davis, California, United States of America; 4 Department of Entomology, Iowa State University, Ames, Iowa, United States of America; 5 Department of Immunology and Microbiology, Scripps Research, La Jolla, California, United States of America; 6 Scripps Research Translational Institute, La Jolla, California, United States of America; University of Alberta, CANADA

## Abstract

It has been 20 years since West Nile virus first emerged in the Americas, and since then, little progress has been made to control outbreaks caused by this virus. After its first detection in New York in 1999, West Nile virus quickly spread across the continent, causing an epidemic of human disease and massive bird die-offs. Now the virus has become endemic to the United States, where an estimated 7 million human infections have occurred, making it the leading mosquito-borne virus infection and the most common cause of viral encephalitis in the country. To bring new attention to one of the most important mosquito-borne viruses in the Americas, we provide an interactive review using Nextstrain: a visualization tool for real-time tracking of pathogen evolution (nextstrain.org/WNV/NA). Nextstrain utilizes a growing database of more than 2,000 West Nile virus genomes and harnesses the power of phylogenetics for students, educators, public health workers, and researchers to visualize key aspects of virus spread and evolution. Using Nextstrain, we use virus genomics to investigate the emergence of West Nile virus in the U S, followed by its rapid spread, evolution in a new environment, establishment of endemic transmission, and subsequent international spread. For each figure, we include a link to Nextstrain to allow the readers to directly interact with and explore the underlying data in new ways. We also provide a brief online narrative that parallels this review to further explain the data and highlight key epidemiological and evolutionary features (nextstrain.org/narratives/twenty-years-of-WNV). Mirroring the dynamic nature of outbreaks, the Nextstrain links provided within this paper are constantly updated as new West Nile virus genomes are shared publicly, helping to stay current with the research. Overall, our review showcases how genomics can track West Nile virus spread and evolution, as well as potentially uncover novel targeted control measures to help alleviate its public health burden.

## Background

West Nile virus (WNV; family Flaviviridae; genus *Flavivirus*) is globally distributed and maintained by a complex transmission cycle involving multiple species of mosquitoes and birds [1–3]. Since its emergence in the Americas in 1999, the virus has resulted in over 48,000 reported cases, 24,000 reported neuroinvasive cases, over 2,300 deaths [4], and an estimated 7 million total human infections in the continental US [5]. At present, WNV is considered one of the most important zoonotic diseases of concern to the US population [6]. WNV is also a significant animal pathogen, having caused over 28,000 reported equine cases [7] and mortality in over 300 bird species [2], resulting in massive population declines reported among at least 23 bird species [8,9]. This includes a reported 45% decline in the American Crow (*Corvus brachyrhynchos*) population following the introduction of WNV [9]. The impact of WNV has not been limited to the US, as more than 5,000 human infections have been reported in Canada [10], and the virus is recognized as an emerging threat across the Americas [11–13].

In the two decades since WNV became established in the US, limited progress has been made in controlling transmission of the virus. Thus, renewed investment in understanding the fundamental aspects of endemic WNV transmission and innovative research directions are needed to mitigate the public health burden of WNV for the next 20 years. For example, advances in virus sequencing and phylogenetic analysis (comparing genetic variation over space and time) have transformed our ability to map the spread and evolution of viruses [14,15]. Such “genomic epidemiology” approaches, for example, were recently deployed to reconstruct the emergence of Zika virus in the Americas [16–18] and revealed the sources causing the yellow fever outbreaks in Brazil [19,20]. For WNV, virus genomics played a central role in uncovering how the virus emerged and became endemic in a new region after its introduction in the US (e.g., [21–27]), and more detailed studies may identify transmission networks and source areas of potential reemergence to be targeted during future interventions [28,29]. While powerful, these phylogenetic tools require technical expertise and computing resources that make them out of reach for some groups. To fill this need, Nextstrain (nextstrain.org) [30] was created to provide everyone the ability to explore pathogen spread and evolution using genomic epidemiology. In this review, we use Nextstrain to uncover key epidemiological and evolutionary processes during the emergence and establishment of WNV in the Americas (nextstrain.org/WNV/NA) and discuss collaborative opportunities to advance this work for future outbreak control.

## Visualizing spread and evolution using Nextstrain

Nextstrain (nextstrain.org) is an open-source initiative to use pathogen genome data to provide a real-time interactive view of the spread and evolution of significant human pathogens [30]. The concept was born from a large collaborative project to track influenza virus evolution using rapid virus sequencing and data sharing [31]. This aided the seasonal influenza vaccine selection process by identifying emerging virus strains that may have evolved resistance to previous vaccines [32,33]. Moreover, recent epidemics of Zika and Ebola have highlighted the need for not only real-time virus genome sequencing but also rapid dissemination of epidemiologically relevant results [34]. Thus, Nextstrain was created to provide a publicly accessible and up-to-date overview of pathogen spread and evolution during outbreaks, facilitated through collaborations with subject matter experts. Alongside platforms such as Virological (virological.org), Nextstrain has become an important venue to quickly disseminate prepublication results, which is especially critical when the information can lead to public health interventions or improved public understanding [34].

Nextstrain is powered by a collection of open-source bioinformatic tools to curate, analyze, and visualize available virus genomes [30]. These datasets are used to reconstruct the geographic structure and estimated virus spread using incorporated phylogenetic tools [14,15]. To reduce runtimes, Nextstrain leverages TreeTime for the maximum likelihood phylogenetic analysis [35], through which the entire WNV dataset currently presented on Nextstrain (*n* = 2,267 genomes as of June 2019) can be analyzed in under an hour on a modern computer.

We created a WNV Nextstrain resource in 2018 to convey the history of WNV in the Americas and promote new research directions (nextstrain.org/WNV/NA; **S1 Fig**). All of the figures herein were created using Nextstrain and contain live display links in each legend to allow the reader to explore the data. The analysis presented on Nextstrain can be recreated using our build pipeline found at github.com/grubaughlab/WNV-nextstrain, which can be used to analyze any WNV dataset offline. Additionally, we designed a new narrative function in Nextstrain to accompany this review—accessible at nextstrain.org/narratives/twenty-years-of-WNV—which walks users through the epidemiological findings discussed here in a more condensed and interactive format. Mirroring the dynamic nature of such research, Nextstrain is constantly updated as new WNV genomes are shared publicly. Thus, the live display links will always contain the most up-to-date information, helping the readers stay current with the research.

## Emergence of an exotic virus

During the summer of 1999, New York City experienced a remarkably high mortality among crows and several exotic captive birds, including Chilean flamingos (*Phoenicopterus chilensis*) in the Bronx Zoo [36]. Meanwhile, health professionals in Queens observed an unusual peak of unexplained human encephalitis cases, but the connection was not initially made with the increased bird mortality [37]. Early serological investigations pointed to Saint Louis encephalitis virus (SLEV), a mosquito-borne flavivirus endemic to the Americas, as the causative agent of the human encephalitis cases [38]. Electron microscopy investigation of viruses isolated from bird tissues also revealed flavivirus-like particles [21]. However, SLEV is usually not associated with mortality in birds, triggering further investigation of a possible link between the virus causing encephalitis in birds and humans [36]. Sequencing of viruses from bird and human tissues ultimately revealed that the 1999 outbreak was not caused by SLEV but, rather, a virus that had never before been observed in the Americas—WNV [21,36,39].

**Fig 1:**
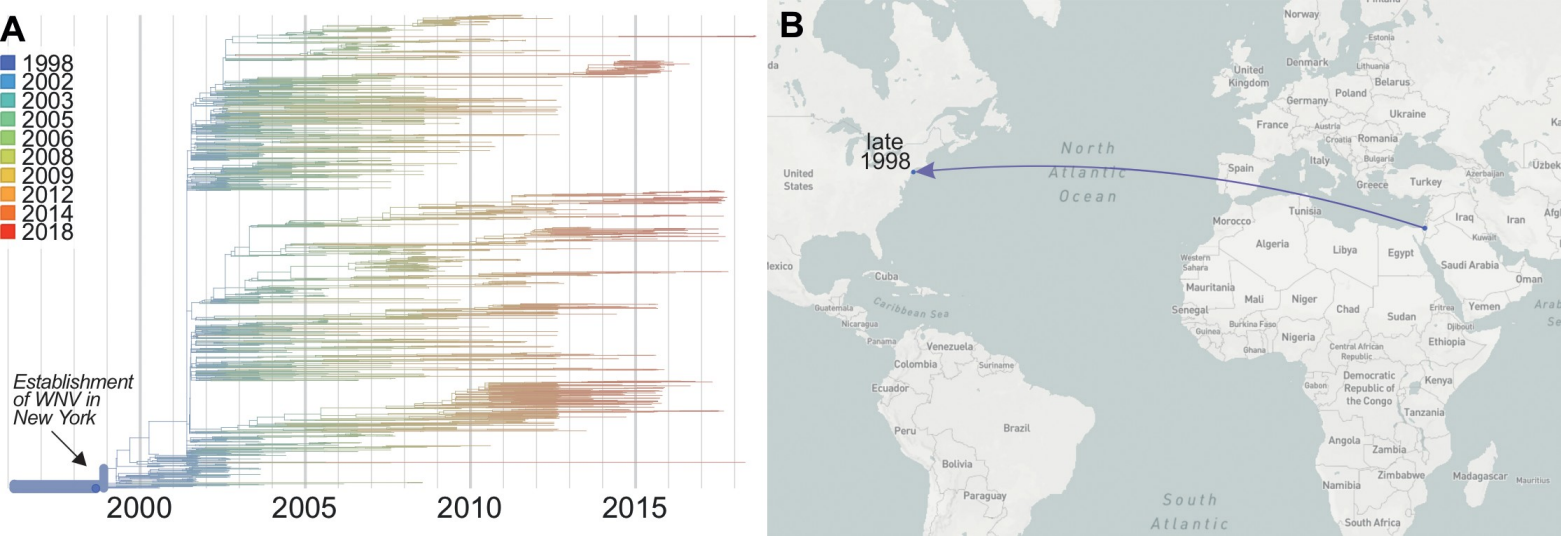
Emergence of WNV in New York. Though WNV was first detected in New York in 1999, (**A**) it likely became established in 1998 (confidence interval = October to December 1998) (**B**) by an introduction possibly from the Middle East, although the exact location cannot be inferred. Data from specific times can be visualized on Nextstrain using the “Date Range” function. A live display can be found at nextstrain.org/WNV/NA?c=num_date&d=tree,map&dmax=1998-12-01&p=grid.

WNV was first isolated in Uganda in 1937 [40] and has since become endemic to many parts of Africa, Europe, Asia, Australia, and the Middle East [41]. Despite its global distribution, WNV was not detected in the Americas before the outbreak in 1999, raising two important questions: (**1**) where did the virus originate from and (**2**) when was it first introduced? Phylogenetic comparisons of the virus sequences isolated from New York in 1999 to WNV sequences from around the world revealed that it was most closely related to a WNV isolate from the brain of a dead goose found in Israel in 1998 [21,39], suggesting a potential origin from the Middle East [42] (**Fig 1**). Further genetic studies revealed that WNV was likely introduced in 1998 [24,43] (**Fig 1**). Thus, WNV may have been circulating in the US for a year before the first outbreak was detected during the summer of 1999.

A still unsettled question is how the virus was brought into the US [44]. The primary mechanism by which dengue and Zika viruses spread around the world is the long-distance travel of infected humans (e.g., [16,45]). However, unlike these viruses, WNV is maintained in a transmission cycle between birds and mosquitoes, in which humans are considered “dead-end” hosts (i.e., do not contribute to the cycle). Therefore, mobility of infected birds or mosquitoes, rather than infected humans, is the more plausible mechanism for the introduction of WNV from the Middle East into the US. While migratory birds might be important dispersers of WNV worldwide [46–48], flyways between the Middle East and North America are not common [3]. A more likely scenario is commercial or unintentional human transportation of birds and/or mosquitoes. Mosquitoes are notorious hitchhikers on airplanes [39,40], making an introduction via one of the two international airports located within the area of the 1999 New York City WNV outbreak a plausible hypothesis. Although it is impossible to determine exactly how WNV was introduced, more detailed future investigations into the patterns of WNV introductions worldwide may reveal new insights.

## Conquering a continent

Following the initial outbreak in New York in 1999 [37], surveillance of mosquitoes and birds showed WNV spread along the eastern seaboard reaching Florida by 2001 [4] and west to the Rocky Mountains and Pacific Northwest (Washington state) by 2002 [4,49]. Finally, in 2003, WNV was detected in Southern California [4,50], marking its successful establishment across the continental US.

**Fig 2:**
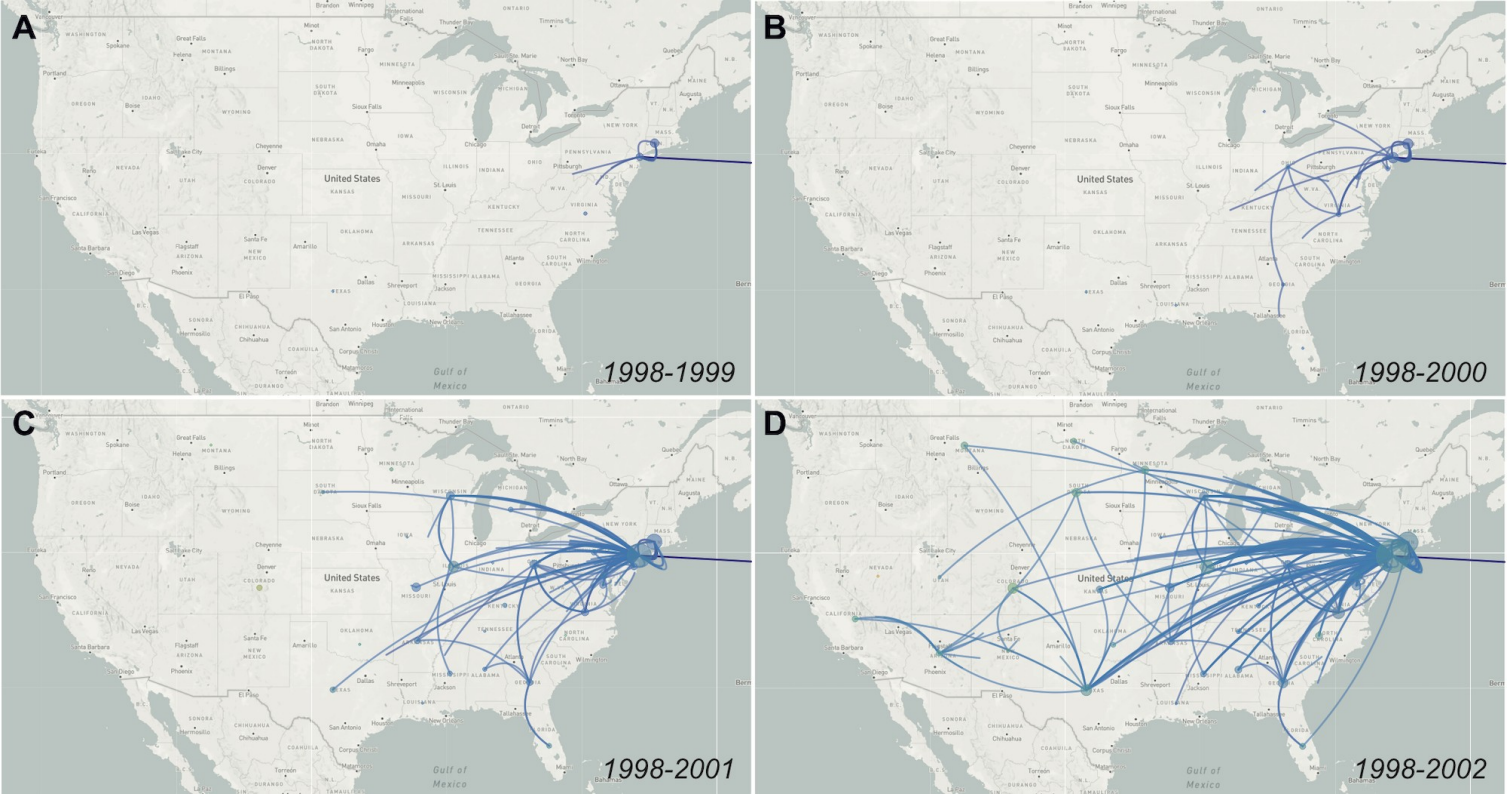
Genomics reveals rapid spread of WNV across the continent. By reconstructing the ancestral traits using TreeTime, Nextstrain infers state of all internal nodes of the phylogenetic tree to map the pattern of WNV spread. The phylogenetic data show that WNV spread to new regions occurred about a year before it was detected by local surveillance. Data from specific times can be visualized on Nextstrain using the “Date Range” function. Live displays can be found at (**A**) nextstrain.org/WNV/NA?c=num_date&d=map&dmax=1999-12-31&f_country=USA, (**B**) nextstrain.org/WNV/NA?c=num_date&d=map&dmax=2000-12-31&f_country=USA, (**C**) nextstrain.org/WNV/NA?c=num_date&d=map&dmax=2001-12-31&f_country=USA, (**D**) nextstrain.org/WNV/NA?c=num_date&d=map&dmax=2002-12-31&f_country=USA.

The virus genomic data reveal that WNV spread even faster across the US than the surveillance data show (**Fig 2**). When combining spatial and phylogenetic data, it is estimated that the WNV “wave” moved from the East Coast to the West Coast at an average dispersal velocity of approximately 1,000 km/year during the first few years (1999–2003) [26]. Moreover, by the time WNV was first detected in New York, the virus had already spread to neighboring states [26] (**Fig 2A**), before reaching parts of the Midwest and Southeast by 2000 (**Fig 2B**). These data also suggest that WNV was established in Texas by 2001 [26] (**Fig 2C**) and California by 2002 [23] (**Fig 2D**), a year before it was detected by local surveillance systems [4,50]. The rapid geographical expansion of WNV between 2001 and 2002 is consistent with a large increase in virus genetic diversity (i.e., a “polytomy,” as a result of many new transmission chains being introduced; **Fig 1A**) [24] and a significant jump in human cases (66 human cases in 2001 to 4,156 in 2002) [4].

During the initial rapid spread of WNV in the US, the lack of geographic structure (i.e., “bush-like’ tree topology; **S2 Fig**) detected from the numerous national [24,26,51–54] and regional [51,55–58] phylogenetic studies suggests that WNV encountered a highly conducive environment with few barriers after it was introduced. This is likely due in part to the large diversity of hosts and vectors that WNV can utilize for transmission [1–3]. Among these, highly abundant passerine birds (e.g., thrushes and sparrows) [59] and *Culex* mosquitoes (e.g., *C*. *tarsalis* and *C*. *pipiens*) [60–62] are the most important for maintaining WNV transmission. Therefore, it is likely that a large population of susceptible hosts and vectors already present in the Americas helped WNV to quickly conquer a continent despite the varied landscapes that it needed to traverse.

While the typical home ranges of residential birds [23,63] and mosquitoes [64] may account for a large portion of WNV dispersal, the rapid rates of spread, nonuniform diffusion pattern, and lack of geographical structure are best explained by frequent mixing of local and nonlocal viruses [26]. Laboratory [65], field [66,67], and phylogenetic studies [24,27] support the hypothesis that the introduction of nonlocal viruses was facilitated by the movement of WNV-infected migratory birds. Indeed, the early spread of WNV along the eastern seaboard aligns with a major bird migration flyway; and while the routes primarily run north and south, the elliptical migration patterns of passerines may account for the East to West WNV expansion [27,48,68]. However, it is also possible that other mechanisms may account for the rapid spread and frequent virus mixing. For example, the impact of human behavior on WNV spread [63], specifically through the unintentional transport of WNV-infected birds or mosquitoes via the dense trucking industry in North America, should be examined more thoroughly. Better understanding of how WNV spreads long distances may be critical to inform future mitigation strategies.

## Aided by evolution?

**Fig 3:**
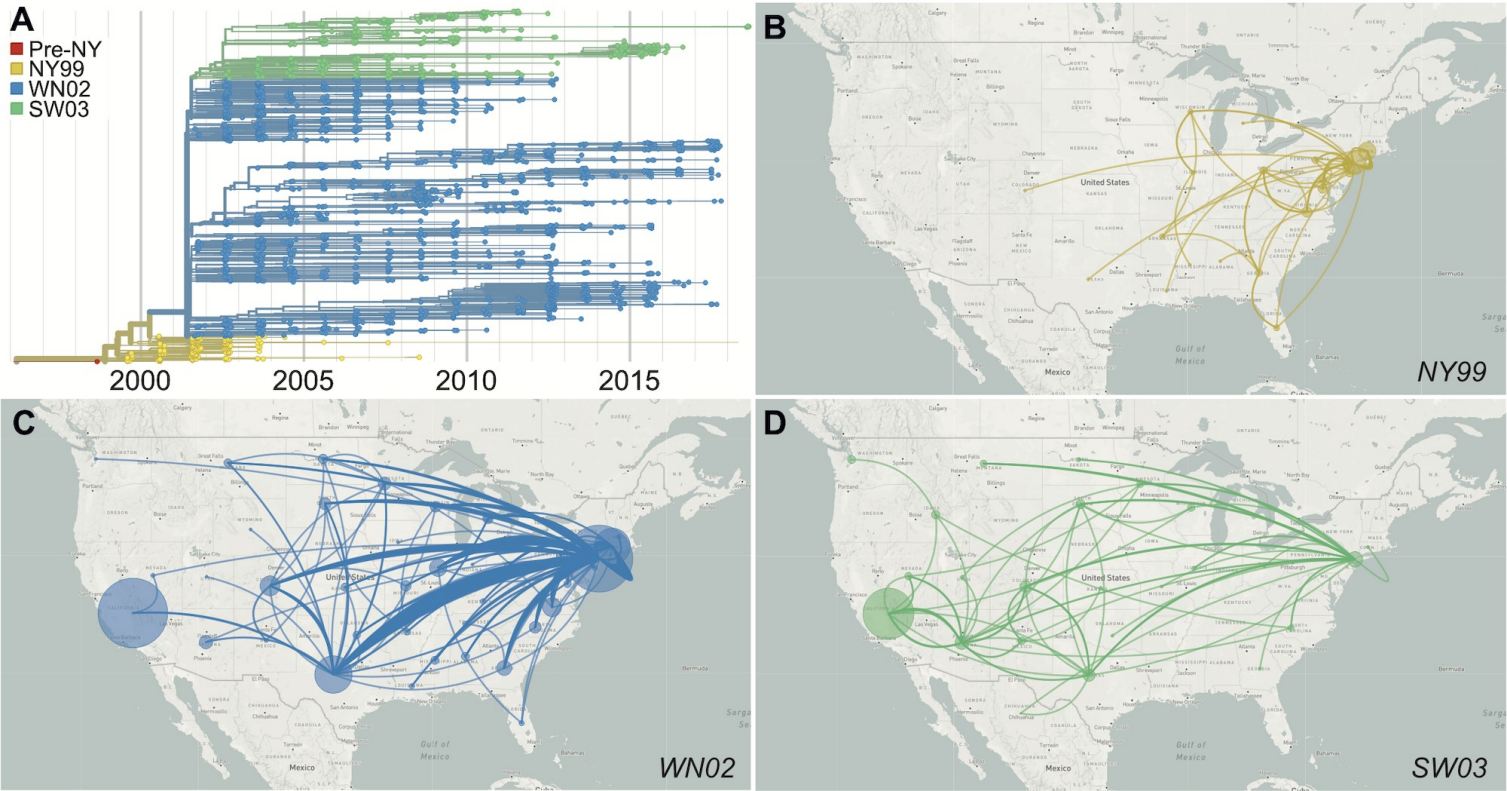
Displacement of the introduced WNV by locally derived genotypes. (**A**) The WNV phylogenetic data support three main genotypes in the US: NY99, the introduced genotype; WN02, defined by the amino acid substitution E-V159A; and SW03, defined by the amino acid substitutions NS4A-A85T and NS5-K314R. The phylogeographic data from the US reveal that (**B**) NY99 rarely migrated past the Mississippi River, while (**C**) WN02 and (**D**) SW03 emerged in the East and spread all the way to the West Coast. WN02 and SW03 continue to cocirculate across the continent. Circle size is relative to the number of genomes that remained local. Data from specific genotypes can be visualized on Nextstrain using the “Color By: Strain” and “Filter by Lineage” functions. Live displays can be found at (**A**) nextstrain.org/WNV/NA?c=lineage&d=tree&f_country=USA, (**B**) nextstrain.org/WNV/NA?c=lineage&d=map&f_country=USA&f_lineage=NY99, (**C**) nextstrain.org/WNV/NA?c=lineage&d=map&f_country=USA&f_lineage=WN02, (**D**) nextstrain.org/WNV/NA?c=lineage&d=map&f_country=USA&f_lineage=SW03.

While WNV continues to diversify, the direct role that evolution played during the emergence of WNV in the Americas is currently difficult to discern. Evolution of WNV and other mosquito-borne viruses is complicated due to their requirement to maintain fitness in alternating and quite disparate mosquito vectors and vertebrate hosts [69–73]. As a result, the WNV evolutionary rate (approximately 4 × 10 ^−4^ substitutions/site/year; **S3 Fig**) is slower than many other single-host RNA viruses [74]; and little evidence for positive selection in the virus population has been detected since the emergence of WNV in the Americas [24]. Yet, the displacement of initial WNV genotype (termed NY99) by locally derived genotypes (WN02 and SW03) [25,51,55,75,76] suggests that the virus may have undergone adaptive evolution in the US (**Fig 3**).

The defining feature of the NY99 genotype displacement is a single amino acid substitution in the WNV envelope protein (E-V159A) that emerged around 2001 [25,51,76] (**Fig 3A**). By 2003, the WN02 genotype containing the E-V159A substitution became dominant (**Fig 3A**), corresponding to the westward spread of the virus (**Fig 3B and 3C**). Experimental studies have shown that compared to NY99 strains, WN02 strains may be more efficiently transmitted by *C*. *pipiens* and *C*. *tarsalis* mosquitoes [76–78] and produce higher viremias during infection of house sparrows (*Passer domesticus*) [79]. Other studies, however, have not found differences in mosquito transmission rates between the genotypes [80–82] and evidence for selection of this allele was not found during phylogenetic [24] or experimental evolution studies [83,84]. Thus, the events leading to the extinction of NY99, either by stochastic processes or through an unknown selective disadvantage, remain unknown.

Soon after the emergence of WN02 in 2001, a southwest genotype (SW03), defined by two amino acid substitutions NS4A-A85T and NS5-K314R, arose from within this clade [24,85] (**Fig 3A**). Both of these substitutions have occurred independently multiple times (**S4 Fig**) and appear to be under positive selection [85,86]. WN02 and SW03 continue to coexist within the US (**Fig 3**) and even cocirculate within the same locations, years, and mosquito vectors (e.g., Arizona [22], California [23], and New York [87]; **S5 Fig**), suggesting that there are not any major barriers segregating these genotypes. However, there is some evidence that SW03 has a more rapid rate of spread than WN02 in California [23]. This could suggest that NS4A-A85T and/or NS5-K314R are the result of recent adaptations; however, the direct fitness of either allele has yet to be experimentally evaluated. Altogether, the evidence that supports the hypothesis that rapid evolution of WNV facilitated its westwardly spread is limited, and additional studies are required to tease apart if locally derived genotypes, such as WN02 and SW03, are specifically adapted to the environment in the Americas.

## Becoming an entrenched virus

Following its emergence, WNV quickly established endemicity and became one of the most important mosquito-borne viruses in North America [4,6]. Since 2002, between 662 (in 2009) and 9,438 (in 2003) human WNV cases have been reported every year in the US [4]. Evidence for endemicity from the genomic data is clear in New York, with some local transmission chains (i.e., branches on the phylogenetic tree) persisting for at least 10 years (**S6 Fig**) [87]. Likely due to the constant repopulation of susceptible mosquito vectors and avian hosts, the data indicate that WNV poses as an annual public health threat with no signs of remission.

Though WNV transmission is consistently detected throughout the continental US, its burden is not uniform, with a large variation in year-to-year human cases and the highest incidence rates often occurring in the central great plains (e.g., Wyoming, South Dakota, and North Dakota) [4]. Dynamic extrinsic factors, such as rainfall and temperature, that influence mosquito and bird populations can be predictive of WNV intensity [88–94], yet the contributions of these extrinsic factors vary across the US due to differences in regional ecology [3,95,96]. This is supported by the nonuniform abundance of *C*. *tarsalis*, *C*. *pipiens*, and *C*. *quinquefasciatus* across the US [97,98], where the presence or absence of these primary mosquito vectors in a given location is mediated by many factors including human land use, elevation, and winter temperatures. Together, these ecological differences create unique transmission networks at the local and regional levels that can significantly influence the likelihood of human disease risks [96–100]. These ecological barriers may further influence mosquito genetic diversity [64,101], causing potential differences in their efficiency for WNV transmission [95,102]. Combined with varied avian ecology and migratory bird flyways across the US [3,96], these data suggest that WNV is maintained in regional transmission networks with unique selective pressures that influence the stability and emergence of new WNV strains [87].

The slowed dispersal velocity and formation of geographical segregation [22–24,26,87] (**S2 Fig, S6 Fig**) after the initial rapid spread stage (**Fig 2**) reinforce the hypothesis that WNV is now primarily maintained at local or regional levels with less long-distance mixing. However, it is unclear if dynamic clustering of the virus across the US will lead to the emergence of new adaptive genotypes and if locally adapted viruses are more likely to cause outbreaks. Understanding such fundamental questions of the epidemiology and biology of WNV strains between and within states may be important for effectively combating this entrenched virus.

## An international concern

The spread of WNV north to Canada and as far south as Argentina highlights that the growing burden of WNV is not limited to the US. The first human WNV case in Canada was reported in Ontario in 2002, and it has now become endemic in several southern Canadian provinces [10,103]. Surveillance between 2001 and 2004 showed that WNV had spread to Central America and the Caribbean [12,13,104–107]. Since then, evidence of WNV transmission has been found across South America, including Colombia, Venezuela, Brazil, and Argentina [13,108–111]. Detection of WNV antibodies in migratory birds suggests that this route may be an important factor for WNV dissemination throughout the Americas [106]; but as described above, it is still unclear if other mechanisms may influence the spread of WNV to Central and South America.

**Fig 4:**
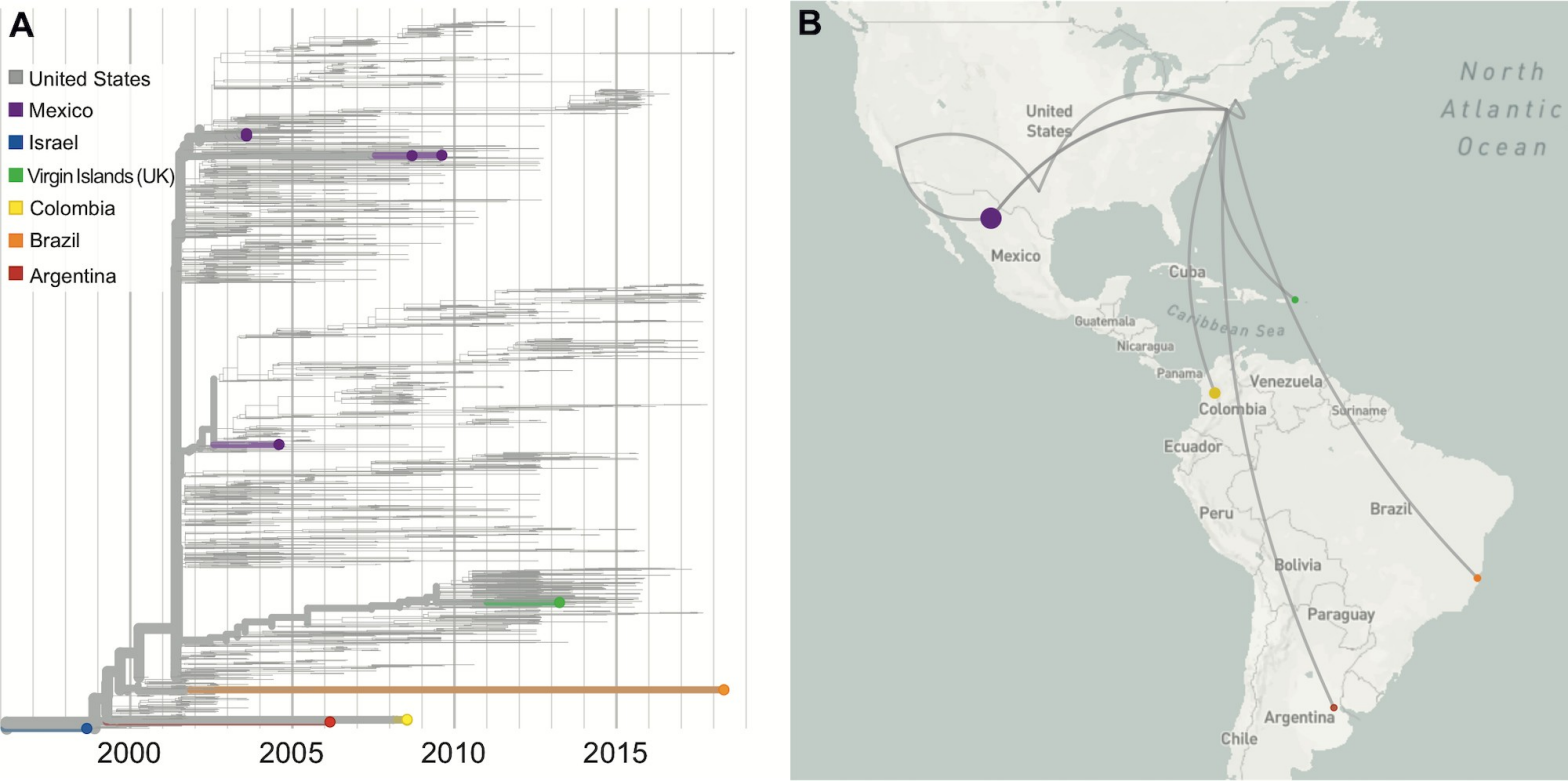
International spread of WNV following its emergence in the US. Following the emergence of WNV in the US, the virus was detected throughout the Americas, from Canada to Argentina. (**A**) The colored branches on the tree correspond to (**B**) the lines on the map depicting spread. However, the limited number of available near-complete WNV genomes from each location and the long branches make it difficult to accurately map the patterns of spread and timing of introductions (e.g., the long orange branch on the tree may indicate that the virus may have traveled to many locations before being introduced in Brazil sometime between 2002 and 2018). Data from specific countries can be visualized on Nextstrain using the “Color By: Country” and “Filter by Country” functions. A live display can be found at nextstrain.org/WNV/NA?c=country&f_country=Argentina,Brazil,British-Virgin-Islands,Colombia,Mexico&p=grid.

Although many partial WNV genomes (primarily envelope coding sequences) were generated from the Americas outside of the US (e.g., [52,112]), complete or near-complete virus genomes are necessary to accurately reconstruct spread and outbreak dynamics [113]. To this end, there are currently only 11 WNV genomes available from Mexico [114,115], seven from the rest of Latin America and the Caribbean [116–119], and none from Canada. These limited data do reveal that the southwards spread into Latin America and the Caribbean began during the early stages of the outbreak in the US and occurred independently multiple times [115–120] (**Fig 4**). In Mexico, multiple introductions of the WN02 and SW03 genotypes suggest that viruses may be commonly moving across the US–Mexico border [114,115]. Moreover, WNV genetic data from the British Virgin Islands [119] and Puerto Rico (partial sequences [121]) reveal that at least the WN02 genotype was introduced into the Caribbean. Interestingly, the NY99 genotype that was displaced in the US (**Fig 3**) was detected in Colombia, Argentina, and Brazil, likely via separate introductions, and may still be in circulation in South America [116–118]. However, the paucity of complete WNV genomes makes it difficult to understand the patterns of spread and diversity outside of the US.

An important question is why large human WNV outbreaks have not been detected in Latin American countries [12,122]. The tropical and subtropical regions of the Americas have suitable conditions for the establishment of WNV, including warm temperatures, diverse avian populations, and a variety of *Culex* mosquitoes [11,122]. Moreover, SLEV, a similar flavivirus maintained by *Culex* vectors and avian hosts is endemic throughout the Americas [123]. Considering that the “old” NY99 genotype virus was recently detected in Brazil [118] and serological evidence of WNV is prevalent in resident birds from Latin America [13,104–106], it is likely that WNV is endemic throughout the Americas. Perhaps the presence of other mosquito-borne viruses has thus far masked human cases (e.g., misdiagnosed as dengue) or may convey some level of cross-protection [12,122]. Alternatively, as WNV may have initially outcompeted SLEV in the US [124], the presence of competitive SLEV genotypes in Central and South America may have at least temporarily slowed WNV transmission in these regions. Given these uncertainties, initiatives to introduce human and equine serosurveys and dead bird and mosquito surveillance for WNV in regions throughout the Americas are paramount for determining its true burden.

## Future role of genomics

**Fig 5:**
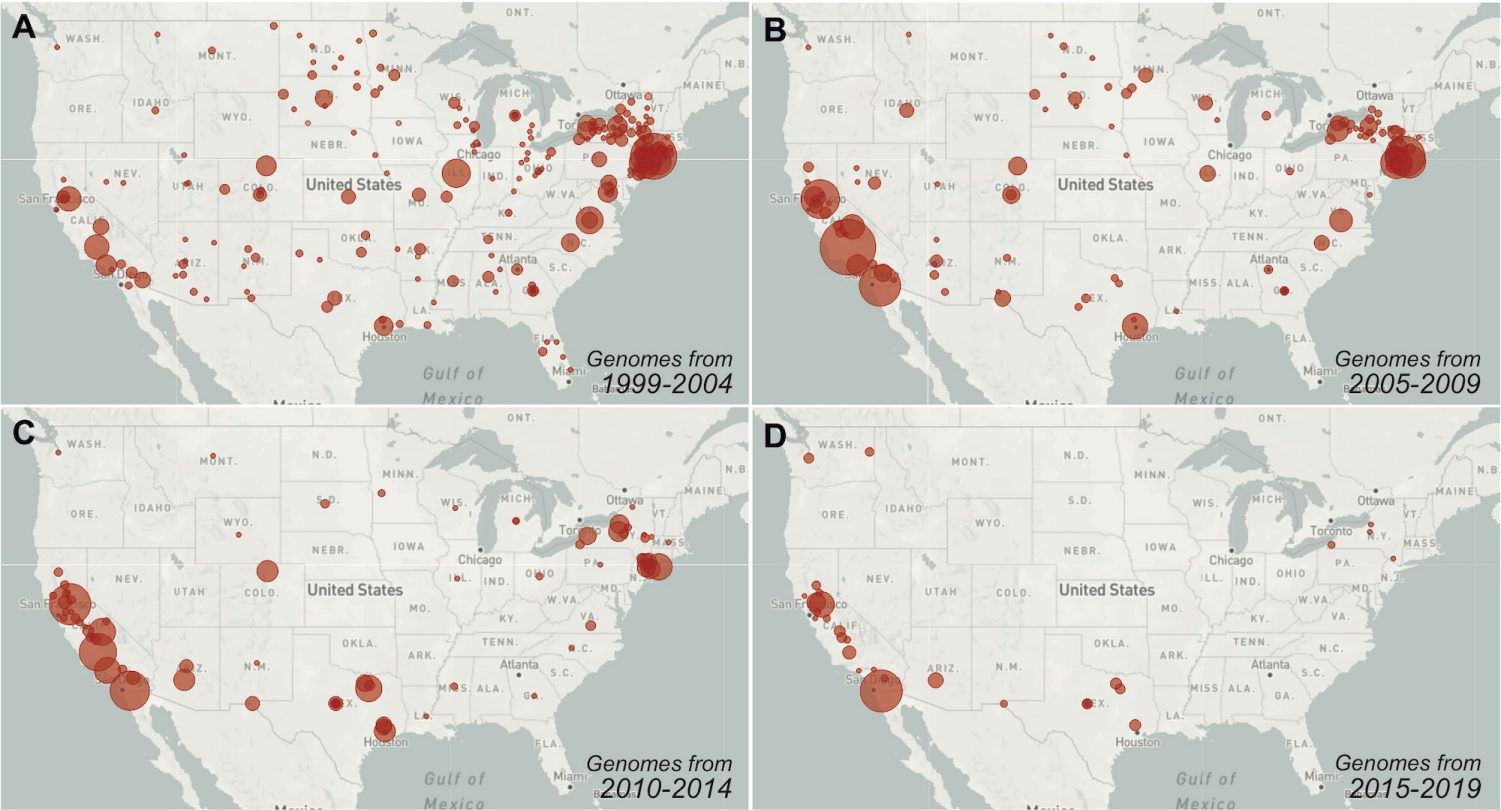
Gaps in recent WNV sampling hinder the usefulness of virus genomic analyses. Following the initial interest in sequencing WNV, the number of available genomes for research has continued to decrease. The circle sizes represent the relative number of near-complete WNV genomes available from each collection location (county) per 5-year period. There are currently (**A**) 728 available WNV genomes from 37 states from 1999 to 2004, (**B**) 789 WNV genomes from 22 from 2005 to 2009, (**C**) 541 WNV genomes from 22 states from 2010 to 2014, and (**D**) 190 WNV genomes from 5 states from 2015 to 2019 (all counts are as of June 2019). Data from specific times can be visualized on Nextstrain using the “Date Range” function. Live displays of the data can be found at (**A**) nextstrain.org/WNV/NA?c=num_date&d=map&dmax=2004-12-31&f_country=USA&r=division, (**B**) nextstrain.org/WNV/NA?c=num_date&d=map&dmax=2009-12-31&dmin=2005-01-01&f_country=USA&r=division, (**C**) nextstrain.org/WNV/NA?c=num_date&d=map&dmax=2014-12-31&dmin=2010-01-01&f_country=USA&r=division, (**D**) nextstrain.org/WNV/NA?c=num_date&d=map&dmax=2019-12-31&dmin=2015-01-01&f_country=USA&r=division.

Given how much WNV has thrived in the US, significant national investment will be required to control future outbreaks. Among this investment should include sustained and collaborative efforts to fill in the many gaps in WNV genomic sampling throughout the country, especially from the last 10 years (**Fig 5**), to better understand endemic WNV transmission using virus genomics approaches [14]. Fortunately, the availability of scalable, easy, and relatively inexpensive sequencing tools (e.g., [125,126]) make these efforts more achievable, and recent work by Swetnam and colleagues [27], Hepp and colleagues [22], Bialosuknia and colleagues [87], and the WestNile 4K Project (westnile4k.org/) provides a template for future public health and academic lab partnerships. Platforms such as Nextstrain (nextstrain.org/WNV/NA) and Virological (virological.org) allow these new data to be continually communicated to the public.

Generating a well-distributed temporal and spatial WNV genomic dataset can be used to determine which strains are causing outbreaks and, importantly, where those strains came from and when they became locally established. This work will help to identify if WNV outbreaks in Ames, Iowa, for example, are connected to outbreaks as far away as Des Moines, Iowa (approximately 60 km); Omaha, Nebraska (approximately 270 km); or even Chicago, Illinois (approximately 560 km). Systematically searching for connections among WNV outbreaks around the country may reveal endemic “transmission networks,” which could be exploited for control and forecasting purposes. WNV control is primarily based on local interventions to temporarily reduce the populations of mosquito vectors [127], but the long-term effects are likely limited by recolonization of mosquitoes and reintroductions of viruses once the pressure is removed. Thus, a highly coordinated effort among several health departments and mosquito abatement districts to synchronize vector control within a transmission network may reduce reintroductions and limit WNV transmission beyond a single season. In addition, genomic epidemiology approaches with large-scale efforts to collect and share mosquito abundance and infection rate data [128] may help to identify potential patterns of virus outbreaks (e.g., as used to identify factors contributing to the Ebola epidemic [29,129]). This, in turn, could be used to maximize vector control efforts by strategically focusing resources at a precise time and location to limit potential outbreaks [29]. After 20 years of WNV in the Americas, it is time to recognize that alleviating the burden of WNV for the next generation will likely depend on significant investment, new approaches, and large collaborations.

## Supporting information

S1 Fig Nextstrain as a tool to visualize WNV evolution and spread. A depiction of the main interactive interface on Nextstrain to visualize the virus (**A**) phylogeny (time-resolved phylogenetic tree created with TreeTime, states listed by two-letter abbreviations, countries listed by three-letter abbreviations), (**B**) spread (inferred patterns of spread based on the tree, circle size is relative to the number of genomes that remained local), and (**C**) genetic diversity (amino acid diversity plotted as entropy and positioned by codon sequence). In the WNV genome, the protein abbreviations are as follows: C = capsid, prM = premembrane, E = envelope, NS1-5 = nonstructural protein 1–5. A live display, which can be used to select and zoom in on various features, can be found at nextstrain.org/WNV/NA?p=grid. WNV, West Nile virus.
(PDF)

S2 Fig Limited geographic structure from early in the epidemic suggests that there were few barriers to WNV spread. An unrooted maximum likelihood WNV phylogenetic tree shows that there is little geographic structure (i.e., bush-like topology) during the early spread phase, as best demonstrated by the Texas (gold) and New York (purple) genomes often clustering together. This suggests that environment was highly conducive for transmission and that the virus could freely spread. The minimal mixing between California and New York genomes, however, suggests that geographic structure is starting to form as the virus transitioned into its endemic phase. Additional WNV sequencing throughout the US from the last 10 years will help to better understand if structure is starting to form and at what scale. States are listed by two-letter abbreviations and countries are listed by three-letter abbreviations. This “unrooted” tree view can be visualized on Nextstrain by toggling between the “Tree Options: Layout”. A live display can be found at nextstrain.org/WNV/NA?l=unrooted&m=div&p=full. WNV, West Nile virus.
(PDF)

S3 Fig Evolutionary rate of WNV in the Americas. A root-to-tip plot showing the divergence (substitutions per site) of sequenced WNV genomes (tips) from the inferred ancestral sequence (root) by the collection dates (shown in years) is used to estimate the evolutionary rate at approximately 4×10^−4^ substitutions/site/year. The tips are colored by increasing time. This “clock” view can be visualized on Nextstrain by toggling between the “Tree Options: Layout.” A live display can be found at nextstrain.org/WNV/NA?c=num_date&d=tree&l=clock. WNV, West Nile virus.
(PDF)

S4 Fig Multiple independent occurrences of genotype-defining WNV mutations. WNV genotype SW03 is defined by two amino acid substitutions, (**A**) nonstructural protein 4A (NS4A) A85T and (**B**) NS5-K314R. Branches (inferred ancestral genome) and tips (sequenced genome) are colored by amino acid at position (**A**) NS4A site 85 (nucleotide position 6721) and (**B**) NS5 site 314 (nucleotide position 8621). Both (**A**) NS4A-A85T and (**B**) NS5-K314R help to form a well-supported clade (yellow branches at the top) but also occur independently throughout the tree (yellow scattered throughout the lower half). All other alleles (nucleotide and amino acid changes) can be visualized using Nextstrain by entering the loci using the “Color By: genotype” function or by selecting a loci on the “Diversity” plot (i.e., **S1C Fig**). Live displays can be found at (**A**) nextstrain.org/WNV/NA?c=gt-NS4A_85 and (**B**) nextstrain.org/WNV/NA?c=gt-NS5_314. WNV, West Nile virus.
(PDF)

S5 Fig Cocirculation of WNV genotypes WN02 and SW03. Since the emergence of WN02 (blue) and SW03 (green) in 2001, the genotypes continue to cocirculate in locations throughout the US For example, both genotypes were detected during the same years and vector species (**A**) in Maricopa County, Arizona [22] (**B**), throughout California [23], and (**C**) throughout New York [87]. Data from other studies can be visualized on Nextstrain by using the “Filter by Authors” function. Live displays can be found at (**A**) https://nextstrain.org/WNV/NA?c=lineage&f_authors=Hepp%20et%20al, (**B**) https://nextstrain.org/WNV/NA?c=lineage&f_authors=Duggal%20et%20al, and (**C**) https://nextstrain.org/WNV/NA?c=lineage&f_authors=Shabman%20et%20al. WNV, West Nile virus.
(PDF)

S6 Fig Establishment of persistent local WNV transmission networks demonstrates endemicity. WNV is likely establishing persistent local transmission networks throughout the US, but this can be easily demonstrated from the 570 genomes available from New York (most generated by [87]). Multiple co-occurring transmission chains (branches) exist derived from either local evolution or separate (re-)introductions, but several persist locally for 5 or more years indicating that those viruses became established. Data from other states can be visualized on Nextstrain by using the “Filter by State” function. A live display can be found at nextstrain.org/WNV/NA?f_state=NY&d=tree. WNV, West Nile virus.
(PDF)
