# Supplementary material for: Twenty years of West Nile virus spread and evolution in the Americas visualized by Nextstrain
Source: PLoS Pathog. 2019 Oct 31;15(10):e1008042. doi: 10.1371/journal.ppat.1008042 (PMC6822705; doi:10.1371/journal.ppat.1008042)
Supplement: S1 Fig — A depiction of the main interactive interface on Nextstrain to visualize the virus (A) phylogeny (time-resolved phylogenetic tree created with TreeTime, states listed by two-letter abbreviations, countries listed by three-letter abbreviations), (B) spread (inferred patterns of spread based on the tree, circle size is relative to the number of genomes that remained local), and (C) genetic diversity (amino acid diversity plotted as entropy and positioned by codon sequence). In the WNV genome, the protein abbreviations are as follows: C = capsid, prM = premembrane, E = envelope, NS1-5 = nonstructural protein 1–5. A live display, which can be used to select and zoom in on various features, can be found at nextstrain.org/WNV/NA?p=grid. WNV, West Nile virus. (PDF) [file ppat.1008042.s001.pdf]

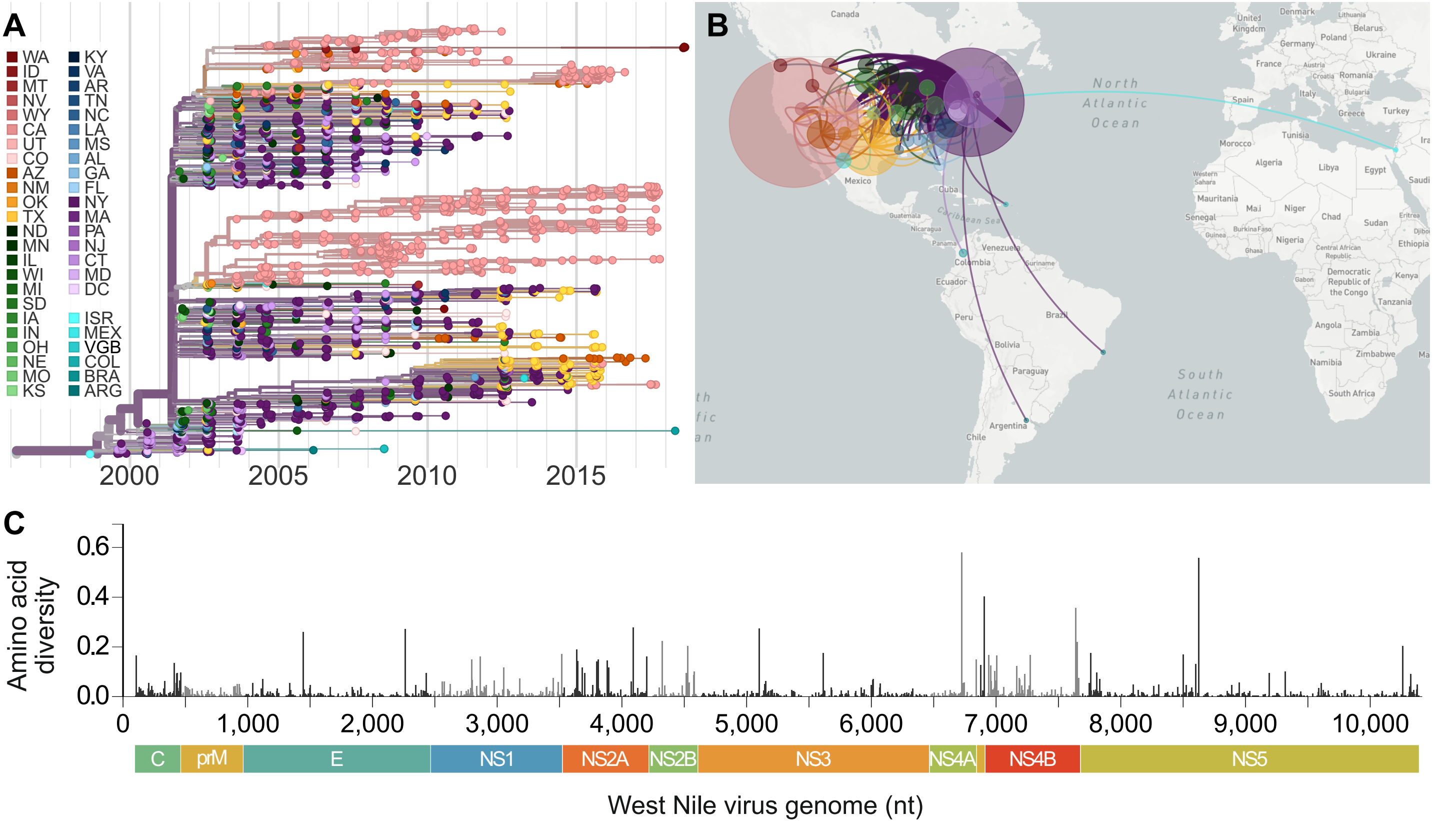

Figure S1. Nextstrain as a tool to visualize West Nile virus evolution and spread. A depiction of the main interactive interface on Nextstrain to visualize the virus (A) phylogeny (time-resolved phylogenetic tree created with TreeTime, states listed by two-letter abbreviations, countries listed by three-letter abbreviations), (B) spread (inferred patterns of spread based on the tree, circle size is relative to the number of genomes that remained local), and (C) genetic diversity (amino acid diversity plotted as entropy and positioned by codon sequence). In the WNV genome, the protein abbreviations are: C = capsid, prM = pre-membrane, E = envelope, NS1-5 = nonstructural protein 1-5. A live display, which can be used to select and zoom in on various features, can be found at: nextstrain.org/WNV/NA?p=grid.
