# Supplementary material for: Twenty years of West Nile virus spread and evolution in the Americas visualized by Nextstrain
Source: PLoS Pathog. 2019 Oct 31;15(10):e1008042. doi: 10.1371/journal.ppat.1008042 (PMC6822705; doi:10.1371/journal.ppat.1008042)
Supplement: S2 Fig — An unrooted maximum likelihood WNV phylogenetic tree shows that there is little geographic structure (i.e., bush-like topology) during the early spread phase, as best demonstrated by the Texas (gold) and New York (purple) genomes often clustering together. This suggests that environment was highly conducive for transmission and that the virus could freely spread. The minimal mixing between California and New York genomes, however, suggests that geographic structure is starting to form as the virus transitioned into its endemic phase. Additional WNV sequencing throughout the US from the last 10 years will help to better understand if structure is starting to form and at what scale. States are listed by two-letter abbreviations and countries are listed by three-letter abbreviations. This “unrooted” tree view can be visualized on Nextstrain by toggling between the “Tree Options: Layout”. A live display can be found at nextstrain.org/WNV/NA?l=unrooted&m=div&p=full. WNV, West Nile virus. (PDF) [file ppat.1008042.s002.pdf]

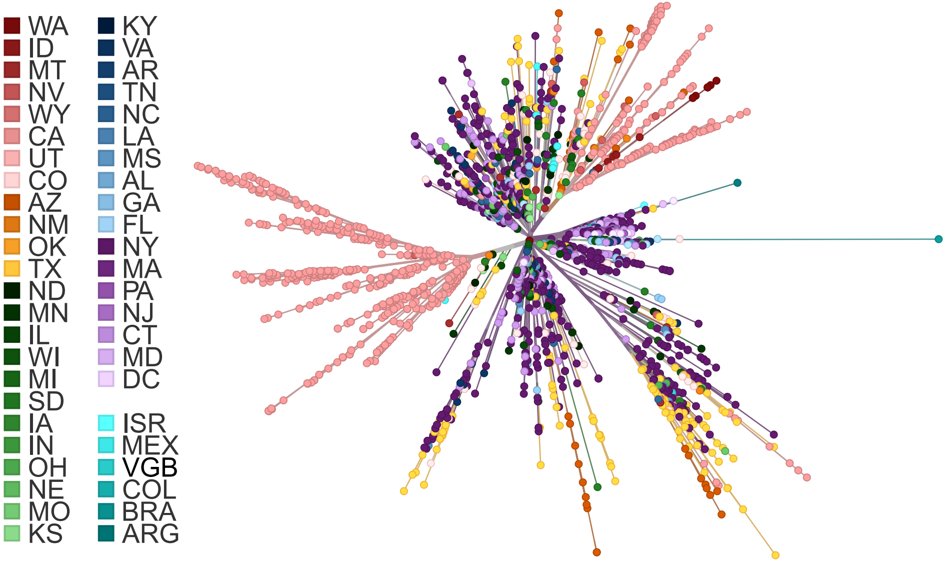

Figure S2. Limited geographic structure early in the epidemic suggests that there were few barriers to West Nile virus spread. An unrooted maximum likelihood WNV phylogenetic tree shows that there is little geographic structure (i.e. 'bush-like' topology) during the early spread phase, as best demonstrated by the Texas (gold) and New York (purple) genomes often clustering together. This suggests that environment was highly conducive for transmission and that the virus could freely spread. The minimal mixing between California and New York genomes, however, suggests that geographic structure is starting to form as the virus transitioned into its endemic phase. Additional WNV sequencing throughout the U.S. from the last 10 years will help to better understand if structure is starting to form and at what scale. States listed by two-letter abbreviations, countries listed by three-letter abbreviations. This "unrooted" tree view can be visualized on Nextstrain by toggling between the "Tree Options: Layout". A live display can be found at: nextstrain.org/WNV/NA?l=unrooted&m=div&p=full.
