# Supplementary material for: Twenty years of West Nile virus spread and evolution in the Americas visualized by Nextstrain
Source: PLoS Pathog. 2019 Oct 31;15(10):e1008042. doi: 10.1371/journal.ppat.1008042 (PMC6822705; doi:10.1371/journal.ppat.1008042)
Supplement: S3 Fig — A root-to-tip plot showing the divergence (substitutions per site) of sequenced WNV genomes (tips) from the inferred ancestral sequence (root) by the collection dates (shown in years) is used to estimate the evolutionary rate at approximately 4×10^−4^ substitutions/site/year. The tips are colored by increasing time. This “clock” view can be visualized on Nextstrain by toggling between the “Tree Options: Layout.” A live display can be found at nextstrain.org/WNV/NA?c=num_date&d=tree&l=clock. WNV, West Nile virus. (PDF) [file ppat.1008042.s003.pdf]

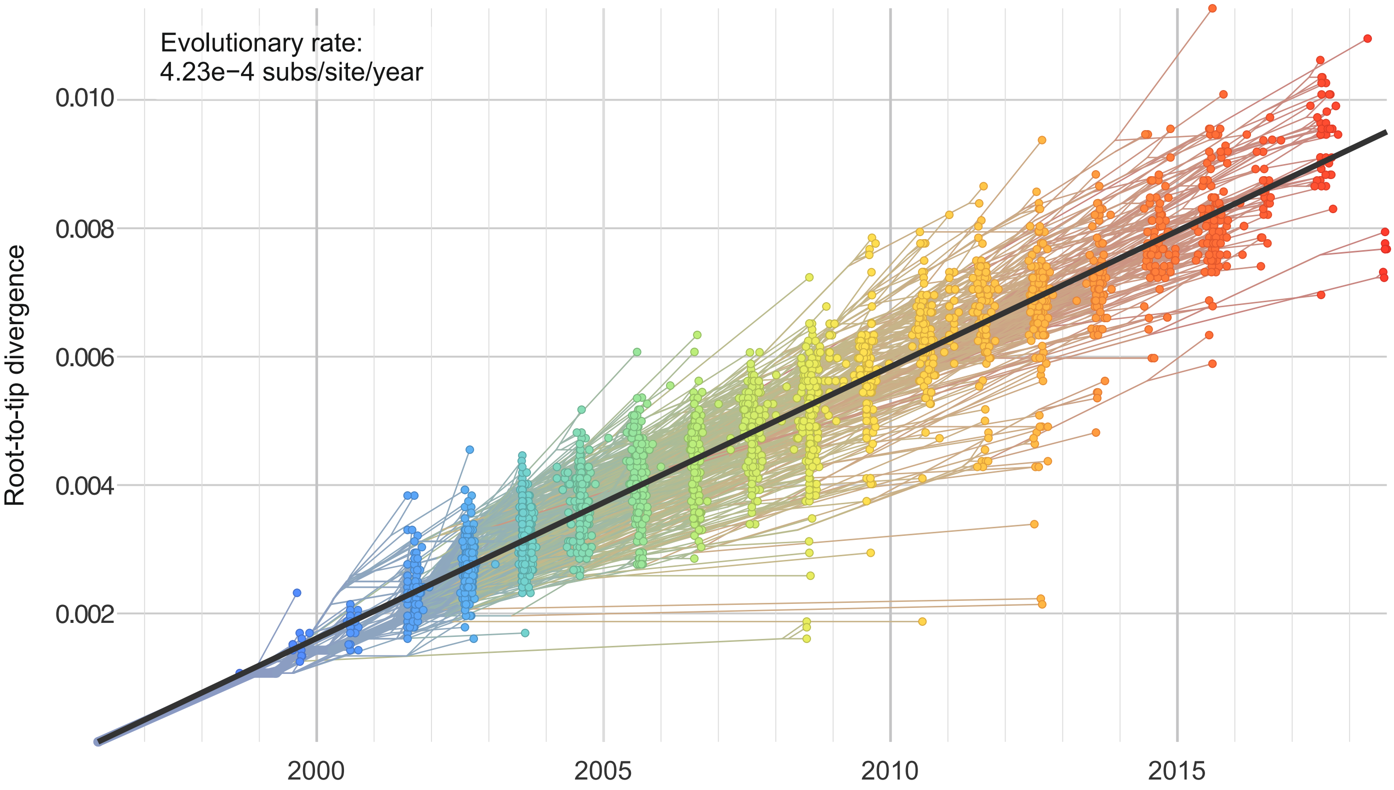

Figure S3. Evolutionary rate of West Nile virus in the Americas. A root-to-tip plot showing the divergence (substitutions per site) of sequenced WNV genomes (tips) from the inferred ancestral sequence (root) by the collection dates (shown in years) is used to estimate the evolutionary rate at ~4×10^{-4} substitutions/site/year. The tips are colored by increasing time. This "clock" view can be visualized on Nextstrain by toggling between the "Tree Options: Layout". A live display can be found at: nextstrain.org/WNV/NA?c=num_date&d=tree&l=clock.
