# Supplementary material for: Twenty years of West Nile virus spread and evolution in the Americas visualized by Nextstrain
Source: PLoS Pathog. 2019 Oct 31;15(10):e1008042. doi: 10.1371/journal.ppat.1008042 (PMC6822705; doi:10.1371/journal.ppat.1008042)
Supplement: S4 Fig — WNV genotype SW03 is defined by two amino acid substitutions, (A) nonstructural protein 4A (NS4A) A85T and (B) NS5-K314R. Branches (inferred ancestral genome) and tips (sequenced genome) are colored by amino acid at position (A) NS4A site 85 (nucleotide position 6721) and (B) NS5 site 314 (nucleotide position 8621). Both (A) NS4A-A85T and (B) NS5-K314R help to form a well-supported clade (yellow branches at the top) but also occur independently throughout the tree (yellow scattered throughout the lower half). All other alleles (nucleotide and amino acid changes) can be visualized using Nextstrain by entering the loci using the “Color By: genotype” function or by selecting a loci on the “Diversity” plot (i.e., S1C Fig). Live displays can be found at (A) nextstrain.org/WNV/NA?c=gt-NS4A_85 and (B) nextstrain.org/WNV/NA?c=gt-NS5_314. WNV, West Nile virus. (PDF) [file ppat.1008042.s004.pdf]

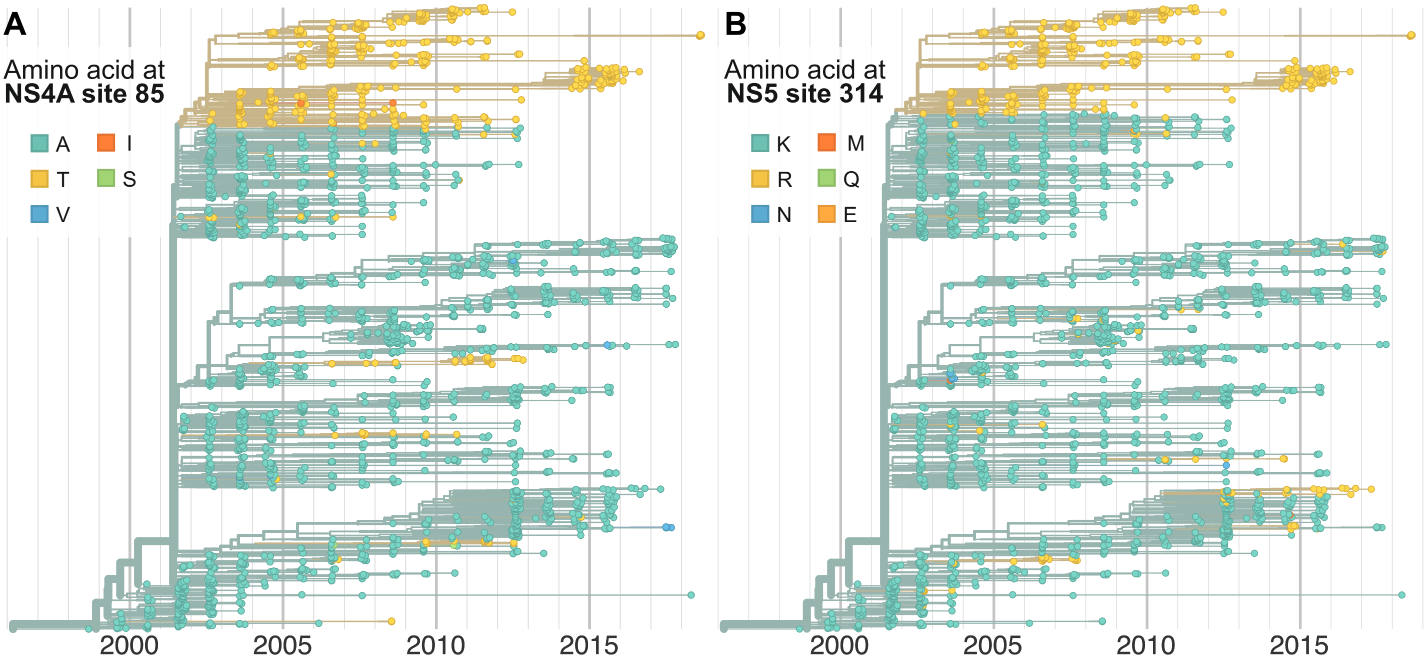

Figure S4. Multiple independent occurrences of genotype-defining West Nile virus mutations. WNV genotype SW03 is defined by two amino acid substitutions, (A) non-structural protein 4A (NS4A) A85T and (B) NS5-K314R. Branches (inferred ancestral genome) and tips (sequenced genome) are colored by amino acid at position (A) NS4A site 85 (nucleotide position 6721) and (B) NS5 site 314 (nucleotide position 8621). Both (A) NS4A-A85T and (B) NS5-K314R help to form a well supported clade (yellow branches at the top), but also occur independently throughout the tree (yellow scattered throughout the lower half). All other alleles (nucleotide and amino acid changes) can be visualized using Nextstrain by entering the loci using the "Color By: genotype" function or by selecting a loci on the "Diversity" plot (i.e. Figure S1C). Live displays can be found at: (A) nextstrain.org/WNV/NA?c=gt-NS4A_85 and (B) nextstrain.org/WNV/NA?c=gt-NS5_314.
