# Supplementary material for: Twenty years of West Nile virus spread and evolution in the Americas visualized by Nextstrain
Source: PLoS Pathog. 2019 Oct 31;15(10):e1008042. doi: 10.1371/journal.ppat.1008042 (PMC6822705; doi:10.1371/journal.ppat.1008042)
Supplement: S5 Fig — Since the emergence of WN02 (blue) and SW03 (green) in 2001, the genotypes continue to cocirculate in locations throughout the US For example, both genotypes were detected during the same years and vector species (A) in Maricopa County, Arizona [22] (B), throughout California [23], and (C) throughout New York [87]. Data from other studies can be visualized on Nextstrain by using the “Filter by Authors” function. Live displays can be found at (A) https://nextstrain.org/WNV/NA?c=lineage&f_authors=Hepp%20et%20al, (B) https://nextstrain.org/WNV/NA?c=lineage&f_authors=Duggal%20et%20al, and (C) https://nextstrain.org/WNV/NA?c=lineage&f_authors=Shabman%20et%20al. WNV, West Nile virus. (PDF) [file ppat.1008042.s005.pdf]

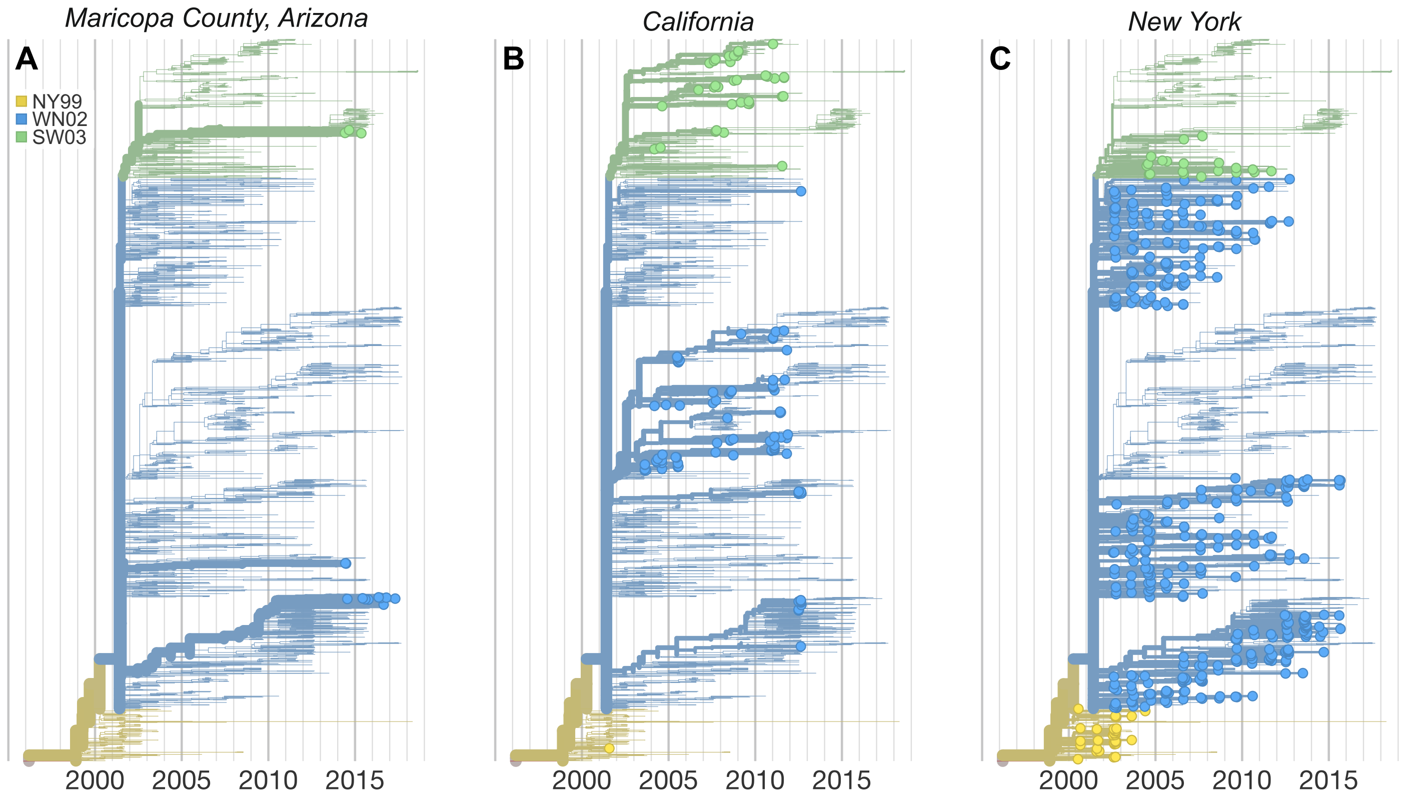

Figure S5. Co-circulation of West Nile virus genotypes WN02 and SW03. Since the emergence of WN02 (blue) and SW03 (green) in 2001, the genotypes continue co-circulate in locations throughout the U.S. For example, both genotypes were detected during the same years and vector species (A) in Maricopa County, Arizona (Hepp et al. 2018) (B) throughout California (Duggal et al. 2015), and (C) throughout New York (Bialosuknia et al. 2019). Data from other studies can be visualized on Nextstrain by using the "Filter by Authors" function. Live displays can be found at: (A) https://nextstrain.org/WNV/NA?c=lineage&f_authors=Hepp%20et%20al
(B) https://nextstrain.org/WNV/NA?c=lineage&f_authors=Duggal%20et%20al, and
(C) https://nextstrain.org/WNV/NA?c=lineage&f_authors=Shabman%20et%20al.
