# Supplementary material for: Twenty years of West Nile virus spread and evolution in the Americas visualized by Nextstrain
Source: PLoS Pathog. 2019 Oct 31;15(10):e1008042. doi: 10.1371/journal.ppat.1008042 (PMC6822705; doi:10.1371/journal.ppat.1008042)
Supplement: S6 Fig — WNV is likely establishing persistent local transmission networks throughout the US, but this can be easily demonstrated from the 570 genomes available from New York (most generated by [87]). Multiple co-occurring transmission chains (branches) exist derived from either local evolution or separate (re-)introductions, but several persist locally for 5 or more years indicating that those viruses became established. Data from other states can be visualized on Nextstrain by using the “Filter by State” function. A live display can be found at nextstrain.org/WNV/NA?f_state=NY&d=tree. WNV, West Nile virus. (PDF) [file ppat.1008042.s006.pdf]

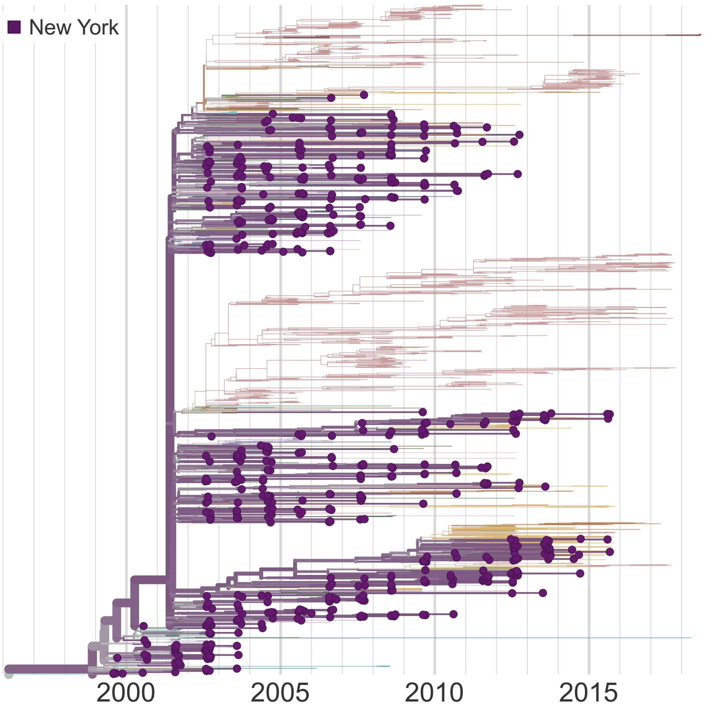

Figure S6. Establishment of persistent local West Nile virus transmission networks demonstrates endemicity. WNV is likely establishing persistent local transmission networks throughout the U.S., but this can be easily demonstrated from the 570 genomes available from New York (most generated by (Bialosuknia et al. 2019)). Multiple co-occurring transmission chains (branches) exist derived from either local evolution or separate (re-)introductions, but several persist locally for 5 or more years indicating that those viruses became established. Data from other states can be visualized on Nextstrain by using the “Filter by State” function. A live display can be found at: nextstrain.org/WNV/NA?f_state=NY&d=tree.
